# Small Extracellular Vesicles in Pre-Therapy Plasma Predict Clinical Outcome in Non-Small-Cell Lung Cancer Patients

**DOI:** 10.3390/cancers13092041

**Published:** 2021-04-23

**Authors:** Eleni-Kyriaki Vetsika, Priyanka Sharma, Ioannis Samaras, Alexandra Markou, Vassilis Georgoulias, Theresa L. Whiteside, Athanasios Kotsakis

**Affiliations:** 1 School of Medicine, University of Crete, 71003 Heraklion, Greece; ekvetsika@med.uoa.gr (E.-K.V.); georgoul@med.uoc.gr (V.G.); 2 School of Medicine, National and Kapodistrian University of Athens, 11527 Athens, Greece; 3 Cancer Center, Department of Pathology, University of Pittsburgh School of Medicine and UPMC Hillman, Pittsburgh, PA 15213, USA; priyahpu@gmail.com (P.S.); whitesidetl@upmc.edu (T.L.W.); 4 Department of Medical Oncology, University General Hospital of Larissa, 41334 Larissa, Greece; jnsamaras@gmail.com (I.S.); alexanmarkou@gmail.com (A.M.); 5 Faculty of Medicine, School of Health Sciences, University of Thessaly, 41334 Larissa, Greece

**Keywords:** small extracellular vesicles, prognostic factor, non-small-cell lung cancer, immunosuppression

## Abstract

**Simple Summary:**

Since re-biopsy of the primary tumor or metastatic lesions is not an option in most NSCLC patients, the use of the “liquid tumor biopsy” represents a promising non-invasive diagnostic/prognostic approach. Small extracellular vesicles (sEV) are currently emerging as a promising source for liquid tumor biopsies. The present study reports that pre-therapy total sEV protein (TEP) levels are associated with patient clinical outcome. We show that the high sEV TEP levels in pre-treatment plasma are a significant negative predictor of PFS and OS in treatment-naïve NSCLC patients. We also observed a positive correlation between the sEV TEP levels and the percentages of circulating CD8^+^PD-1^+^ and CD8^+^PD-L1^+^T cells at baseline prior to any therapy. This suggested that sEV in plasma hinders anti-tumor immune responses via the PD-L1/PD-1 pathway. The data suggest that pre-therapy plasma sEV levels could be useful as non-invasive predictors of NSCLC progression and outcome after conventional therapy.

**Abstract:**

The potential use of plasma-derived small extracellular vesicles (sEV) as predictors of response to therapy and clinical outcome in chemotherapy-naïve patients with non-small-cell lung cancer (NSCLC) was explored. sEV were isolated by size-exclusion chromatography from the plasma of 79 chemotherapy-naïve NSCLC patients and 12 healthy donors (HD). sEV were characterized with regard to protein content, particle size, counts by qNano, morphology by transmission electron microscopy, and molecular profiles by Western blots. PD-1 and PD-L1 expression on circulating immune cells was analysed by flow cytometry. Pre-treatment levels of total sEV protein (TEP) were correlated with overall (OS) and progression-free survival (PFS). The sEV numbers and protein levels were significantly elevated in the plasma of NSCLC patients compared to HD (*p* = 0.009 and 0.0001, respectively). Baseline TEP levels were higher in patients who developed progressive disease compared to patients with stable disease (*p* = 0.007 and 0.001, stage III and IV, respectively). Patient-derived sEV were enriched in immunosuppressive proteins as compared to proteins carried by sEV from HD. TEP levels were positively correlated with CD8^+^PD-1^+^ and CD8^+^PD-L1^+^ circulating T cell percentages and were independently associated with poorer PFS (*p* < 0.00001) and OS (*p* < 0.00001). Pre-therapy sEV could be useful as non-invasive biomarkers of response to therapy and clinical outcome in NSCLC.

## 1. Introduction

Lung cancer is the most common cause of cancer-related death worldwide [1,2]. Non-small-cell lung cancer (NSCLC) accounts for about 85% of cancer-related deaths, with 5-year survival rates of about 2% for unresectable NSCLC when treated with conventional anti-cancer treatments such as surgery, radiotherapy, and chemotherapy [3,4]. Despite the significant therapeutic advances achieved using immunotherapies, the 5-year overall survival (OS) remains below 15% [1]. Since anti-cancer therapies are not beneficial to all NSCLC patients, there is an unmet need for the identification of prognostic and predictive biomarkers in order to optimize patient treatments and facilitate the selection of potentially responsive patients. Given that re-biopsy of the primary tumor or metastatic lesions is not an option in most NSCLC patients, the use of a “liquid tumor biopsy” represents a promising non-invasive diagnostic/prognostic approach.

Small extracellular vesicles (sEV) are currently emerging as a promising source for liquid tumor biopsies, as their presence in plasma has been correlated with tumor progression and immune evasion of cancer [5,6,7,8]. Extracellular vesicles (EVs) are released through various mechanisms by all living cells and are present in all body fluids, including blood, urine, saliva, and ascites [9]. EVs that originate from intraluminal membrane invagination in the multivesicular bodies (MVBs) and are released following fusion of MVBs with the plasma membrane represent a subset of small EVs generally referred to as exosomes. Because these vesicles are heterogeneous in size, comprising exomeres (<30 nm), small exosomes (EXO-S: 30–90 nm), and large exosomes (EXO-L: 90–150 nm) [10], they are currently named “sEV” to distinguish them from larger microvesicles. sEV mediate intercellular communication. They carry lipids, proteins, RNA, and DNA species, and their content recapitulates that of parent cells. In cancer, tumor cells use sEV to transfer proteins and nucleic acids to recipient cells at local or distant body sites. Such information transfer leads to the functional reprogramming of recipient non-malignant cells and alteration of the tumor microenvironment (TME) into one that supports tumor growth, tumorigenesis, angiogenesis, drug resistance, and metastasis [11,12]. Tumor-derived sEV also promote tumor immune escape by interacting with immune effector cells in the TME and the circulation by suppressing anti-tumor functions [13,14].

Total sEV fractions in patient plasma contain both tumor-derived and non-tumor-derived vesicles in various ratios that vary in the individual patients. Therefore, the molecular/genetic cargo that sEV carry can provide significant information about the tumor and might be a useful source of disease biomarkers in cancer patients [15]. Recently, several studies reported that elevated levels of plasma sEV might have diagnostic value in patients with cancer, including those with lung cancer [16,17,18,19,20,21]. Few studies have also evaluated the prognostic significance of the exosomal miRNA or EVs in the blood of NSCLC patients [22,23,24,25] and only one study has shown that the presence of NY-ESO-1^+^exosomes was correlated with poor survival in NSCLC patients [26]. Interestingly, there are no studies, so far, investigating the levels of total sEV protein (TEP) in the blood of NSCLC patients and correlating TEP levels with the response and clinical outcome to therapy. Indeed, studies revealed that high levels of total sEV protein were a predictor of survival in melanoma patients [27], and TEP levels were decreased with a response to anti-cancer therapy in head and neck cancer patients [28,29]. The current study aimed to explore the potential predicting value of the TEP in pre-therapy plasma for response and clinical outcome in chemotherapy-naïve NSCLC receiving first-line treatment.

## 2. Materials and Methods

### 2.1. Patients and Plasma Samples

Seventy-six chemotherapy-naïve NSCLC patients and 12 age- and sex-matched healthy blood donors (HD) were recruited to participate in the study. All patients were older than 18 years old and had not been treated with any immunosuppressive drug or granulocyte colony-stimulating factor before blood collection for this study. The stage of the disease at the time of diagnosis was based on the 7th lung cancer TNM classification and staging system [30], and the Response Evaluation Criteria in Solid Tumors (RECIST) version 1.1 was used to assess the tumor response to therapy [31]. The study was compliant with the Ethical Principles for Medical Research Involving Human Subjects according to the World Medical Association Declaration of Helsinki, and was approved by the local ethics and scientific committees of the University Hospital of Heraklion (Greece), No.17869–16 December 2014 and No.20068–30 January 2015. All patients and HD provided written informed consent for participation in the study.

Peripheral blood (10 mL) was collected into K_2_EDTA (BD Biosciences, Heidelberg, Germany) at the time of diagnosis and before the administration of first-line chemotherapy. Blood samples from HD were used as controls. The blood samples were immediately centrifuged at 1400× *g* for 10 min to obtain plasma and were stored in 1 mL aliquots at −80 °C until used for exosome isolation.

Exosome isolation from plasma was performed using size-exclusion chromatography (SEC). Plasma samples were thawed and then underwent a series of centrifugation steps: first at 2000× *g* for 10 min at 4 °C to remove all cell debris and then at 10,000× *g* for 30 min to remove large microvesicles and platelets. Next, ultrafiltration through a 0.22 µm pre-wetted filter (EMD Millipore, Billerica, MA, USA) was performed and a 1 mL aliquot of pre-clarified plasma was used for sEV isolation by Size-Exclusion Chromatography (SEC) as previously described [32]. Briefly, 1.5 cm × 12 cm mini-columns (Bio-Rad, Hercules, CA, USA; Econo-Pac columns) were packed with 10 mL of Sepharose 2B (Sigma-Aldrich, St. Louis, MO, USA). A porous frit was placed on top of the gel to minimize disturbance during exosome isolation. Each column was washed with 20 mL of PBS, and 1.0 mL of pre-clarified plasma was placed on the mini-SEC column and eluted with PBS. One-milliliter fractions were collected, with sEV eluting in fraction #4. Fraction #4 was enriched in sEV, as previously reported [32].

### 2.2. Protein Measurement Using the BCA Protein Assay

The total protein content of the isolated sEV fractions was measured using the Pierce BCA protein assay kit (Pierce Biotechnology, Rockford, IL, USA) according to the manufacturer’s instructions. sEV protein levels in fraction #4 were normalized to 1 mL of plasma, and the protein concentrations are reported as μg protein/mL of plasma used for sEV isolation by a mini-SEC column.

### 2.3. Exosome Size and Concentration Assessment by Tunable Resistive Pulse Sensing (TRPS)

The size ranges and concentration of isolated sEV in fraction #4 were measured using TRPS as previously described by the authors [32]. Izon software version 3.2 was used for data recording and for calculating nanoparticle size ranges and concentrations.

### 2.4. Transmission Electron Microscopy (TEM)

The morphology of the isolated sEV was assessed using TEM at the Center for Biologic Imaging at the University of Pittsburgh. Freshly isolated exosomes were layered on copper grids with 0.125% formvar in chloroform and stained with 1% uranyl acetate in ddH_2_O. Exosomes were visualized using a transmission electron microscope (JEOL JEM-1011, JEOL Inc., Peabody, MA, USA).

### 2.5. Detection of the Immune Cargo in Isolated sEV by Western Blots

The isolated sEV were concentrated using a 100 kDa Amicon Ultra 0.5 mL centrifugal filter (EMD Millipore, Billerica, MA, USA). sEV were tested for the presence of exosome markers, including TSG101 and other protein markers of interest as shown below. Briefly, samples of sEV (10 µg protein/sample) were lysed in Lane Marker Reducing Sample Buffer (Thermo Scientific, Rockford, IL, USA) and 10 µg of protein were loaded on each lane of a mini-protein TGX pre-cast gel (Bio-Rad, Hercules, CA, USA). The separated proteins were transferred onto Immobilon-P PVDF membranes (EMD Millipore, Billerica, MA, USA). The membranes were incubated overnight at 4 °C with various antibodies (Abs) as follows: cytotoxic T-lymphocyte-associated protein (CTLA-4; 1:500, #ab134090), TNF-related apoptosis-inducing ligand (TRAIL;1:500, #ab2056), Fas (1:1000, #ab133619), Fas ligand (FasL; 1:500, #ab68338), and OX40 ligand (OX40L/CD252; 1:1000, #ab108083) purchased from Abcam (Cambridge, UK); CD39 (1:400, #sc-33558), CD73 (1:400, #sc-25603), and cyclooxygenase-2 (COX-2; 1:500, #sc-1745) obtained from Santa Cruz Biotechnology (Dallas, TX, USA); programmed cell death protein 1 (PD-1; 1:500, #MAB1086), programmed death-ligand 1 (PD-L1; 1:400, #MAB156), and LAP/TGF-β1 (1:10, #AF-246-NA) from R&D (Minneapolis, MI, USA); tumor susceptibility gene 101 protein (TSG101; 1:500, #PA5-31260) from Invitrogen (Thermo Fisher, Waltham, MA, USA); and ALG-2 interacting protein X (ALIX; 1:500, #2171S) from Cell Signaling (Cell Signaling Technology, Danvers, MA, USA). HRP-conjugated secondary antibody (1:3000–1:5000; Thermo Fisher, Waltham, MA, USA) was added for 1 h at room temperature (RT) and blots were developed with ECL detection reagents (GE Healthcare Biosciences, Uppsala, Sweden). Band intensities on exposed films were quantified using Image J software (NIH, Bethesda, MD, USA). The integrated pixel value was determined for each protein band by multiplying the image intensity and the band area after subtracting the mean background value.

### 2.6. Flow Cytometry for Immunophenotypic Analysis and Enumeration of Immune Cells

Blood samples (10 mL) were processed using Red Blood Cell (RBC) Lysing Buffer (BD Biosciences). Freshly isolated white blood cells were studied for the expression of surface markers using the following anti-human fluorochrome-conjugated monoclonal antibodies: anti-PD-1 BV605 (#329924), anti-PD-L1 BV655 (# 563740; BD Biosciences), and (a) for DC/monocytes: anti-CD14 PE Cy7 (# 325618), anti-HLA-DR APC-H7 (#307618), anti-Lin (CD3/CD56/CD19) PE (# 555340/555516/555413, respectively, BD Biosciences), (b) for T cells: anti-CD3 PE/Dazzle 594 (#300450), anti-CD4 BV510 (#317444), anti-CD8APC/Fire750 (#344746), and (c) for B cells: anti-CD19-Pacific Blue (#302232), and anti-CD3 PE-Dazzle 594 (#300450). All antibodies were purchased from Biolegend, San Diego, CA, USA unless otherwise stated. Staining was performed for 30 min on ice in the dark. After washing, cells were resuspended in 0.5 mL FACS buffer. BD LSR II Flow Cytometer (BD Biosciences) and the FACS Diva Software were used for acquisition and multicolor analysis. Unstained cells were used as a negative control to set the gates and 10^6^ single events were collected for each measurement. The analysis of T-cell subsets and B cells was restricted to the lymphocytic population, whereas for DC/monocytes, gates were restricted to the monocytic population.

### 2.7. Statistical Analysis

This was prospective, translational, a single-institution study investigating the clinical relevance of the levels of TEP in patients with NSCLC. Because of the observational nature of the study, it was not possible to propose a statistical hypothesis to estimate the appropriate number of patients to be enrolled in the study. Statistical analysis was performed using GraphPad Prism version 6.0 (GraphPad Institute Inc., San Diego, CA, USA). Data are presented as means ± SEM. The TEP levels were compared between groups using the non-parametric Mann–Whitney test and the parametric unpaired *t*-test. Qualitative factors were compared by Pearson’s chi-squared test or Fisher’s exact test when appropriate. For the progression-free survival (PFS) or overall survival (OS) analysis and the association with the effector immune cells, TEP levels were dichotomized into low and high using the TEP median value of all patients. Median PFS and OS were estimated using the Kaplan–Meier method with groups compared using the log-rank test. The PFS was defined as the time between the enrolment and the date of first clinical progression or death. The OS was defined as the time from the study enrollment to death. Univariate and multivariate Cox regression hazards models were performed using the SPSS Statistics 23 software (SPSS Inc., Chicago, IL, USA). Differences and associations were considered significant when *p* < 0.05. All *p* values were two-sided.

## 3. Results

### 3.1. Clinicopathological Characteristics of NSCLC Patients

Between March 2011 and January 2017 (March 2011 and January 2017), 76 chemotherapy-naïve patients with newly diagnosed NSCLC were enrolled in the study at the Department of Medical Oncology of University General Hospital in Heraklion, Crete, and had available plasma for sEV isolation. The clinical and pathological patient characteristics are presented in Table 1. The median age was 65 years, 82.9% were men, 51.3% had adenocarcinoma, and 30.3% had stage III and 69.7% had stage IV disease. Samples from 32 (42.1%) patients were screened for EGFR mutations. All patients but one, who refused any treatment, received 3–6 cycles of first-line chemotherapy. Thirty-nine (51.3%) patients achieved an objective response (PR/SD). Seventy-four (97.3%) patients were evaluated for assessment of clinical outcome.

### 3.2. Characterization of Exosomes Isolated from the Plasma of NSCLC Patients

**Figure 1:**
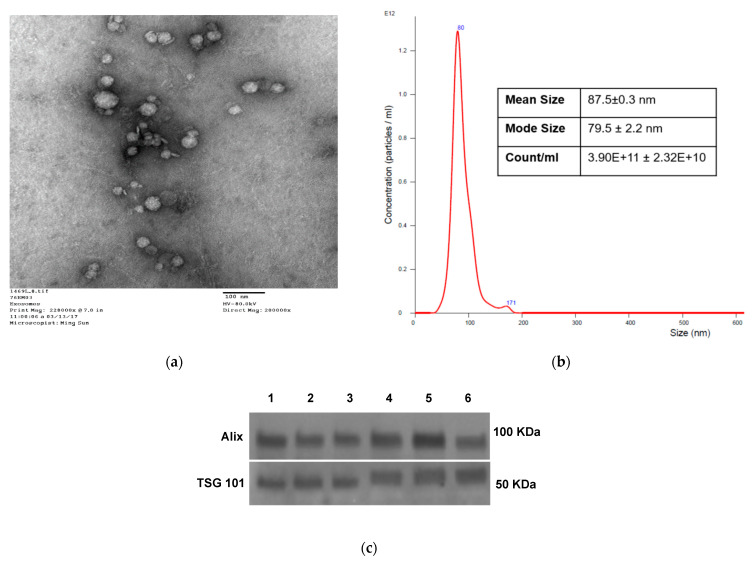
Characteristics of sEV isolated from NSCLC plasma. (**a**) Transmission electron microscopy of isolated NSCLC exosomes. (**b**) Size and concentration of NSCLC exosomes as determined by tunable resistive sensing (TRPS). (**c**) TSG101 and ALIX detection by Western blots in sEV isolated from plasma of several different patients with NSCLC. Each lane was loaded with 10 µg sEV protein present in fraction #4.

Transmission electron microscope (TEM) indicated that the isolated exosomes (fraction #4) from the plasma of chemotherapy-naïve NSCLC patients had a vesicular appearance (Figure 1a) with a diameter of about 50–150 nm, as confirmed by the NanoSight analysis (Figure 1b). Western plots showed that the isolated sEV carried TSG101 and ALIX, markers of the endosomal origin for sEV (Figure 1c). Uncropped Western blot images in Figure S1.

**Figure 2:**
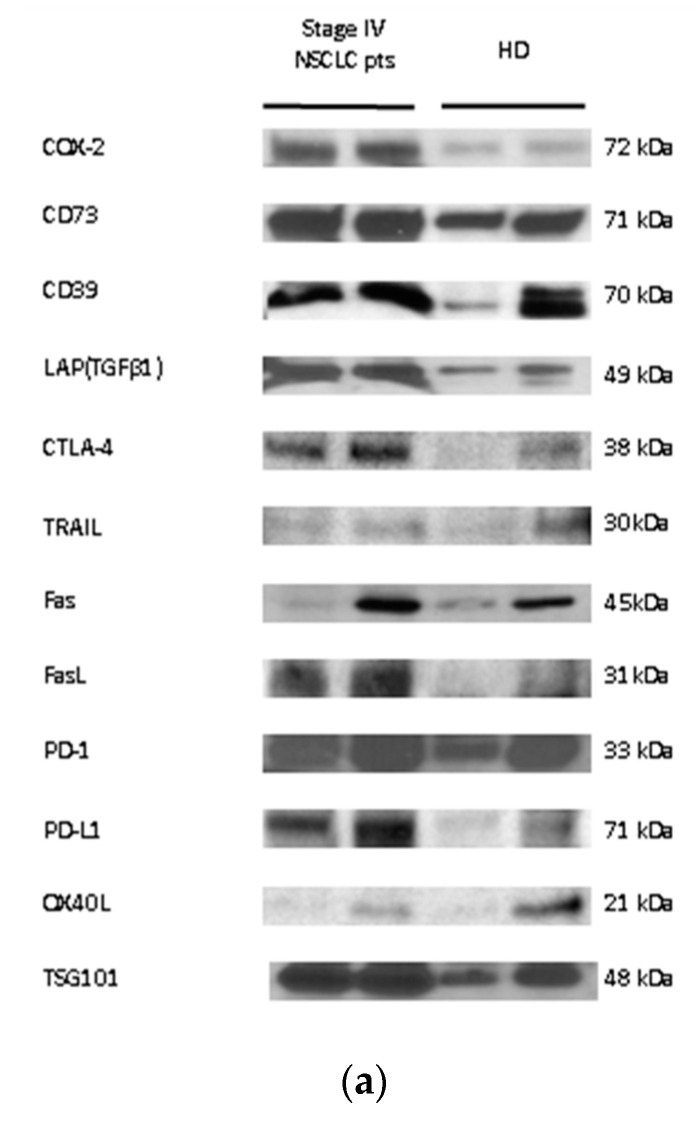

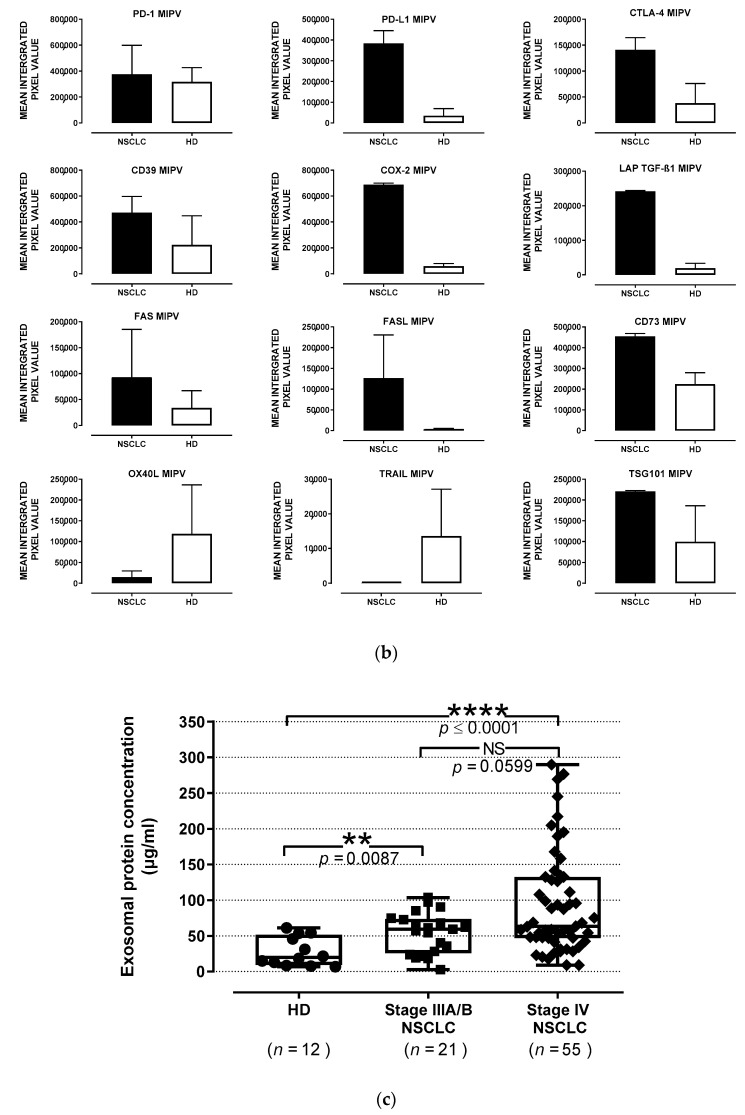
Molecular content and baseline protein levels of sEV isolated from NSCLC plasma. (**a**) Representative Western blot (WB) of sEV isolated from the plasma of NSCLC or HD. Each lane of the WB contained 10 µg exosomal protein. (**b**) Semi-quantitative densitometry of the Western blots as described in Methods. The bars denote mean values ± SEM. (open bars = HD, *n* = 2; closed bars = stage IV NSCLC patients, *n* = 2). (**c**) The exosomal protein levels (μg/mL plasma) in patients were higher compared with HD. Each point corresponds to an individual patient or HD (black circles = HD, black squares = stage IIIa/b NSCLC patients, black rhombus = stage IIIa/b NSCLC patients). The medians, 75th percentile (box), and maximum and minimum (whiskers) are represented. Groups were compared using the Mann–Whitney U test (HD vs. stage IV; stage IIIA/B vs. IV) and unpaired *t*-test (HD vs. stage IIIA/B). (*n*: number of patients; NSCLC: non-small-cell lung cancer; HD: healthy donor; **, *p* < 0.01; ****, *p* < 0.0001; NS: non-significant).

The cargos of sEV isolated from NSCLC patient plasma before the initiation of systemic treatment and sEV isolated from HD plasma were assessed by Western blots (WBs). As indicated in Methods, each lane was loaded with 10 µg of exosomal protein. Selected immunosuppressive or immune-activating proteins were assessed, and TSG-101 served as control of the sEV endocytic origin. Figure 2a shows the presence and enrichment of several immunosuppressive molecules in sEV from the plasma of two patients with stage IV NSCLC relative to sEV from the plasma of two HD. Figure 2b shows the results of a semi-quantitative density analysis of the WBs. The sEV from patients’ plasma carried higher levels of immunosuppressive molecules, including CD39, CD73, PD-1, PD-L1, CTLA-4, TGFβ, Fas, FasL, and COX-2 relative to sEV of HD. The patients’ sEV carried lower levels of OX40L, an immunostimulatory protein, than sEV of HD, and also carried less TRAIL than sEV of HD. This suggests that in NSCLC patients with advanced disease, TRAIL carried by sEV might not have a pro-apoptotic effect due to its binding to the decoy receptors TRAIL-R3 (DcR1) and TRAIL-R4 (DcR2). The semiquantitative densitometry data in Figure 2b are representative of two patients and two HD, and while their significance remains unconfirmed, the observed enrichment in immunosuppressive proteins corresponds to the results previously reported by us for sEV from the plasma of patients with HNC [33,34,35] or melanoma [36,37].

### 3.3. Association of TEP Levels with the Clinicopathological Characteristics of Chemotherapy-Naïve NSCLC Patients

Figure 2c shows that TEP levels of sEV isolated from plasma of chemo-naïve NSCLC patients were elevated (54.27 ± 6.19 μg/mL, stage III, and 93.77 ± 9.67 μg/mL, stage IV) compared to TEP levels of sEV isolated from HD plasma (28.07 ± 5.88 μg/mL; *p* = 0.0087 and <0.0001, respectively). Although the TEP levels were found to be higher in stage IV compared to stage III patients, the difference between these two patient groups was not significant (*p* = 0.06).

The TEP levels of sEV were examined for association with the clinicopathologic data for all NSCLC patients. The TEP levels were dichotomized into low (<61.82 μg/mL) and high (≥61.82 μg/mL) using the median value for all patients. As shown in Table 1, high TEP levels were significantly associated with age (*p* = 0.037) and response to treatment (*p* = 0.001; Table 1).

### 3.4. Prognostic Role of TEP Levels in Patients with NSCLC

**Figure 3:**
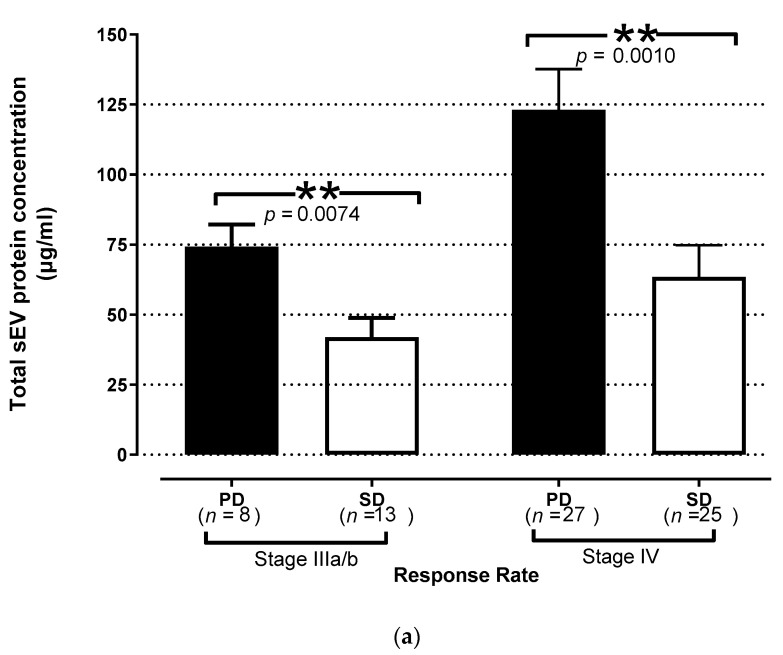

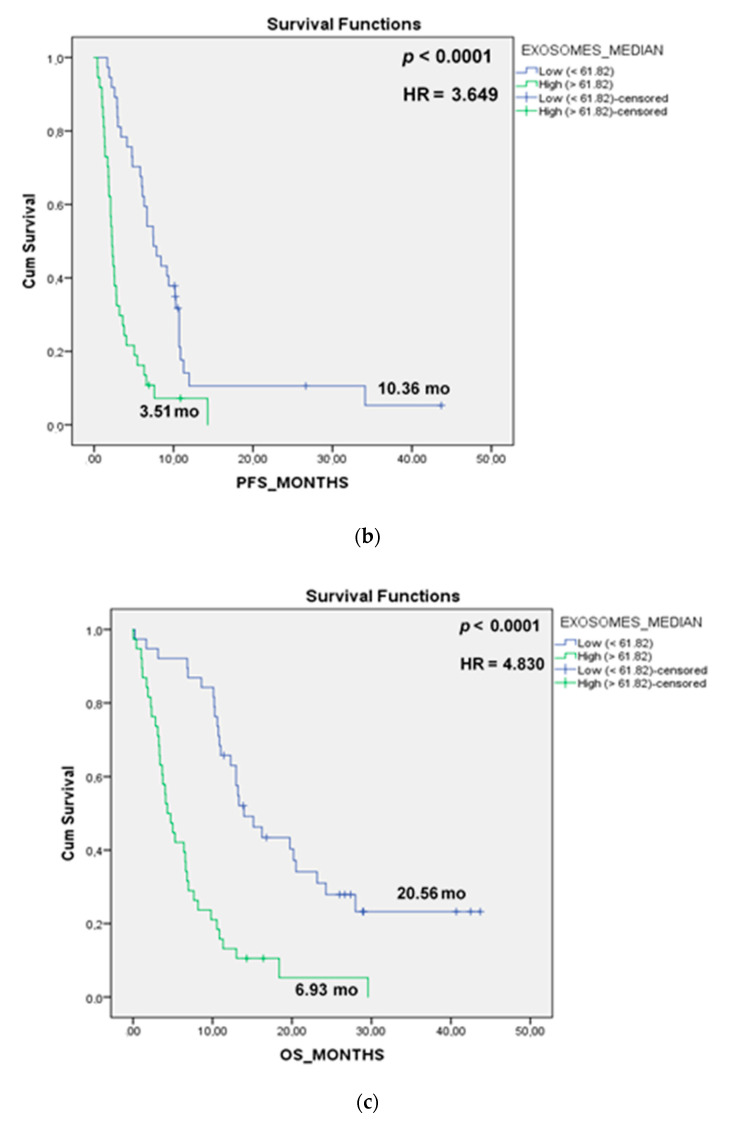
Prognostic significance of sEV in chemotherapy-naïve NSCLC patients. (**a**) Response to treatment in patients according to TEP levels at baseline. The levels were increased in patients with disease progression (PD, closed bars) compared with patients controlling their disease (open bars) after 3 cycles of therapy. The bars denote mean values ± SEM. Kaplan–Meier plots of (**b**) PFS and (**c**) OS in patients according to high and low TEP levels in plasma. The high TEP levels were correlated with worse PFS (*p* < 0.0001) and OS (*p* < 0.0001); blue line: below the median, green line: above the median (n: number of patients; PR: partial response; SD: stable disease; PD: progressive disease; PFS; progression-free survival; OS: overall survival; NSCLC: non-small-cell lung cancer; **, *p* < 0.01).

Patients with either stage III or IV NSCLC experiencing disease progression (PD) after three chemotherapy cycles had significantly increased levels of TEP in plasma at baseline (74.28 ± 7.88 μg/mL, stage IIIA/B, and 123.1 ± 14.54 μg/mL, stage IV) compared to patients who responded to therapy and achieved disease control (DC) (41.96 ± 6.92 μg/mL, *p =* 0.007 and 63.45 ± 11.34 μg/mL, *p* = 0.001, respectively; Figure 3a).

The patients were dichotomized into those with high or low levels, using the patients’ TEP levels median value as the cut-off level to group the patients according to the two possible extreme values. Patients with low TEP levels at baseline experienced longer PFS compared to those with high TEP levels (10.36 vs. 3.51 months, respectively; *p* < 0.0001, Figure 3b). Moreover, high TEP levels were associated with significantly shorter OS (20.56 months vs. 6.93 months, *p* < 0.0001) compared with low levels (Figure 3c).

The univariate Cox proportional hazards regression analysis revealed that high TEP levels (hazard ratio (HR) = 3.074, *p* < 0.0001) and stage IV (HR = 2.223, *p* = 0.005) were significantly correlated with decreased PFS. Moreover, decreased OS was significantly associated with high TEP levels (HR = 3.755, *p* < 0.0001, Table 2). In addition, the multivariate analysis showed that stage IV vs. Stage IIIA/B and increased TEP levels in plasma were independent prognostic factors for decreased PFS (HR = 1.955, *p* = 0.02; HR = 2.852, *p* < 0.0001, respectively), while an increased TEP level was the only independent prognostic factor associated with decreased OS (HR = 3.755, *p* < 0.0001; Table 2).

### 3.5. Quantitative Distribution of Peripheral Immune Effector Cells in NSCLC Patients with High and Low TEP Levels

**Figure 4:**
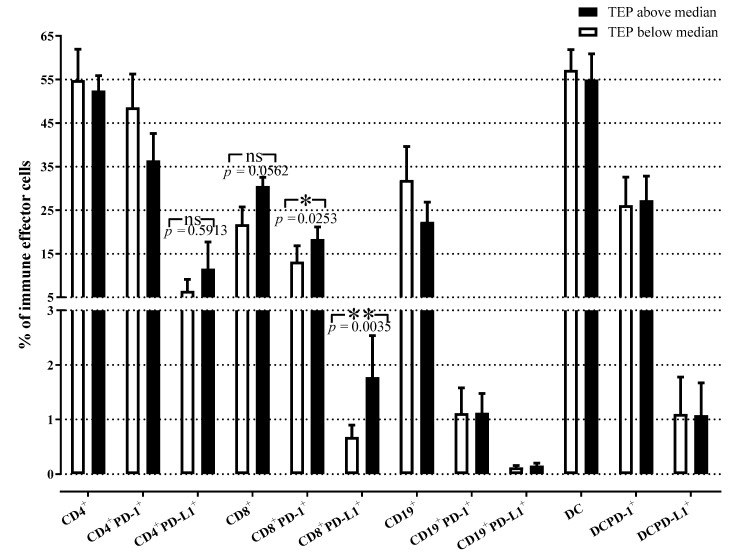
Relationship between TEP levels and peripheral immune cells in patients with NSCLC. Percentages of different effector immune cell subpopulations in stage IV patients are related to the TEP levels at baseline. The percentages of CD8^+^PD-1^+^ and CD8^+^PD-L1^+^T cells were significantly increased in patients with high TEP levels as compared to patients with low TEP levels. The bars denote mean values ± SEM. Groups were compared by Mann–Whitney U test (TEP above vs. below median for CD4^+^PD-L1^+^, CD8^+^PD-L1^+^, CD19^+^PD-L1^+^, DCPD-L1^+^) and unpaired *t*-test (TEP above vs. below median for CD4^+^, CD4^+^PD-1^+^, CD8^+^, CD8^+^PD-1^+^, CD19^+^, CD19^+^PD-1^+^, DC, DCPD-1^+^; open bars = TEP below the median, closed bars = TEP above the median; TEP = total sEV protein; *, *p* < 0.05; **, *p* < 0.01).

To examine a potential relationship between the percentages of circulating effector immune cells and plasma sEV at baseline, stage IV NSCLC patients were divided into two groups according to TEP levels (low vs. high). Interestingly, patients with high TEP levels had significantly increased levels of CD8^+^ T cells expressing the immunosuppressive molecules PD-1 (*p* = 0.03) and PD-L1 (*p* = 0.004) compared to patients with low TEP levels (Figure 4). The percentages of total CD8^+^ T cells were numerically higher in patients with high TEP levels compared to those with low TEP levels; however, this difference was not significant (*p* = 0.056). The percentages of CD4^+^ T cells, B-cells, and DC/monocytes expressing PD-1 or PD-L1 molecules or not, were not significantly different between these two groups of patients. Overall, these results suggested that elevated TEP levels in NSCLC plasma were associated with the increased frequency of circulating CD8^+^PD-L1^+^ effector T cells and perhaps also CD4^+^PD-L1^+^ T cells prior to treatment at baseline. This association implies that reprogramming of T cells by sEV toward immunosuppressive phenotype at baseline might also influence the outcome.

## 4. Discussion

This study evaluated the presence and levels of circulating sEV in pre-therapy plasma and their association with the clinical outcome of newly diagnosed chemotherapy-naive patients with NSCLC. The results demonstrated elevated numbers of sEV and total sEV protein (TEP) levels in the plasma of NSCLC patients compared to HD. Furthermore, among patients with NSCLC, sEV numbers and TEP levels prior to any treatment were higher in patients with stage IV compared to patients with stage IIIA/B disease. Importantly, the high level of circulating sEVwas an independent negative predictive/prognostic factor for PFS and OS in NSCLC patients.

Although the field of sEV has been rapidly growing and sEV are emerging as important players in cancer progression, there is still no “gold-standard” for exosome isolation and quantification [38]. To the best of our knowledge, the present study demonstrates for the first time the isolation of sEV from the plasma of NSCLC using the mini-SEC isolation protocol followed by the BCA method for quantification of the levels of total sEV protein (TEP). The SEC isolation method we used has previously been reported as efficacious in removing considerable (but not all) quantities of “contaminating’ plasma proteins from EVs [32,39]. Since this method utilizes small sample volumes, is rapid, and is amenable to high sample throughput, it can be readily used in studies searching for potential biomarkers in clinical samples. Indeed, using SEC we were able to isolate morphologically intact, functionally-active sEV from the pre-cleared plasma of NSCLC patients and HD, as previously reported [14,32,39]. We found that NSCLC patients had higher levels of sEV in their plasma than HD. However, there was no statistical difference in sEV numbers or TEP between patients with different stages of NSCLC. The current results are in agreement with the data reported in patients with esophageal cancer, where the exosome levels in patients’ plasma were higher compared to patients with non-malignant conditions [40]. 

The discovery and subsequent validation of robust, sensitive, and specific predictive and prognostic biomarkers for NSCLC are of critical importance. Their use could facilitate the selection of patients who most likely will respond to the treatment or those who might need an immune intervention. To date, anti-PD-L1 immunohistochemistry (IHC) on tissue sections has been the only validated companion diagnostic test for first-line immunotherapy for advanced and metastatic NSCLC [41]. However, the detection of this biomarker presents limitations that have stimulated the development of other approaches. For example, the use of a liquid biopsy (LB) could provide an important complementary strategy to PD-L1 IHC. sEV, together with circulating tumor cells (CTC), circulating tumor DNA (ctDNA), and cytokines, is considered as a component of LB [41]. The present study provides support for the role of sEV as LB in NSCLC. We have uncovered the association of pre-therapy TEP levels with the patient’s clinical outcome following standard of care (SOC) therapy. We observed that plasma TEP levels at baseline in patients who experienced disease progression upon three cycles of therapy were significantly increased compared to the TEP levels observed in non-progressors, irrespective of the disease stage. This could be related to the fact that sEV production levels increase with disease progression, whereby the larger tumor load is associated with an increased immunosuppressive cargo [34,42]. Furthermore, the current study demonstrates for the first time the independent predictive and prognostic value of TEP as revealed by multivariate analysis (Table 2). High levels of TEP at baseline before initiation of any systemic treatment were associated with the worst PFS and OS compared to NSCLC patients with low TEP levels. This finding should be interpreted with caution given the relatively low number of patients enrolled in the study. Nevertheless, our observations suggest that baseline levels of sEV represent an independent prognostic factor for PFS and OS in patients with NSCLC. Several studies have investigated the prognostic value of sEV in plasma of patients with NSCLC [23,24,25,43,44,45]. However, these studies were all based on examining specific exosomal proteins or exosomal microRNAs, and none considered total sEV protein concentrations.

An important finding in the current study is the observed positive correlation between the TEP levels and the percentages of the CD8^+^PD-1^+^ and CD8^+^PD-L1^+^T cells at baseline prior to any therapy. Here, this finding is reported for the first time for NSCLC patients in the literature, and provides strong evidence of the immunosuppressive effect of sEV. Previous studies have indicated that sEV play an important role in modulating the host immune response by transferring information from cancer to non-malignant cells and especially by sending negative signals to the immune cells [9,11]. This sEV-driven process, referred to as “reprogramming”, converts immune cells to pro-tumor immunosuppressive phenotype [11]. Tumor-derived exosomes (TEX) are especially effective in modulating functions of all types of immune cells, as previously described [13,14,33]. TEX carrying a cargo of various immunosuppressive proteins deliver inhibitory signals to activated T or NK cells [11,33], which results in (a) inhibition of proliferation and cytolytic activity of CD8^+^ effector T cells; (b) inhibition of NK cell activity; and (c) induction/expansion of suppressive immune cells such as MDSCs and Tregs [13,15]. Indeed, in this study, using WB we found that sEV isolated from the plasma of chemotherapy-naïve NSCLC patients carried higher levels of immunosuppressive molecules such as CD39, CD73, PD-1, PD-L1, CTLA-4, TGFβ, Fas, FasL, and COX-2, and few immunostimulatory molecules (OX40L, TRAIL) when compared to the cargo of sEV originating from HD plasma (Figure 2). This observation strongly suggests that in newly diagnosed, chemotherapy-naive patients with locally advanced and metastatic NSCLC, circulating sEV hinder the anti-tumor immune response.

We showed that the presence of sEV at high levels correlated with increased frequencies of CD8^+^PD-1^+^ and CD8^+^PD-L1^+^T-cells in treatment-naïve patients, suggesting a possible mechanism by which sEV suppress immune stimulation via the PD-L1/PD-1 pathway, eventually leading to the impairment of anti-tumor immune responses. These sEV consist of TEX and also of immune cell-derived exosomes (IEX) produced by immunocytes reprogrammed by TEX. CD8^+^PD-L1^+^T cells secreting IEX carry inhibitory PDL-1^+^and presumably contribute to elevated levels of TEP in these patients. sEV-containing elevated levels of immunosuppressive molecules within TEP are instrumental in reprogramming CD8^+^ T cells from an anti-tumor effector phenotype to an immunosuppressive pro-tumor phenotype. In a previous study, we demonstrated that baseline levels of CD8^+^PD-L1^+^ T cells represent an independent prognostic factor for OS and PFS in patients with stage IV NSCLC [46]. With the data reported here showing that TEP levels at baseline are associated with response to therapy and clinical outcome of NSCLC patients and that sEV contribute to the suppression of immune surveillance, it is reasonable to conclude that sEV levels prior to any therapy are also prognostically significant. The potential impact of PD-L1^+^ sEV on disease activity or responses to immunotherapy has been previously reported [47,48]. Further studies of PD-L1^+^ immune cells and PD-L1^+^ sEV in patients with NSCLC will be of great interest. Considering this direction, we have already initiated the selection of plasma in patients treated with immunotherapy alone or a combination of chemotherapy and immunotherapy.

## 5. Conclusions

The current study indicates that high levels of circulating sEV in therapy-naïve patients with NSCLC are a potential new predictive/prognostic factor for disease outcome after conventional therapy. The fact that sEV carry immunosuppressive molecules strongly suggests that their presence might be of especially high importance for patients undergoing immunotherapy. Therefore, future studies in a larger group of NSCLC patients treated with immune checkpoint inhibitors would help to confirm this notion.

## Figures and Tables

**Table 1:** The patients’ clinicopathological characteristics.

	Patients (*n* = 76) Levels of Exosomes			
Parameters	*n* (%)	Low	High	*p* Value
Age (years)	Median 65 (range, 44–84)			**0.037**
<65	38 (50)	12	22	
≥65	38 (50)	26	16	
Gender				0.544
Male	63 (82.9)	30	33	
Female	13 (17.1)	8	5	
Stage				0.318
Stage IIIA/B	23 (30.3)	14	9	
Stage IV	53 (69.7)	24	29	
Histology				0.100
Squamous	37 (48.7)	19	18	
Non-squamous	39 (51.3)	19	20	
EGFR status				0.484
Wild-type	30 (39.5)	16	14	
Mutant	2 (2.6)	0	2	
NE	44 (57.9)	22	22	
Platinum-based chemotherapy regimen				0.725
Taxanes	35 (47.9)	18	17	
Pemetrexed	18 (24.7)	8	10	
Gemcitabine	18 (24.7)	10	8	
Vinorelbine	2 (2.7)	1	1	
Response to therapy (after 3 cycles)				**0.001**
PR	19 (25)	14	5	
SD	20 (26.3)	15	5	
PD	35 (46)	8	27	
NE	2 (2.7)	1	1	

*n*: number of patients; EGFR: epidermal growth factor receptor, PR: partial response; SD: stable disease; PD: progressive disease; NE: not evaluated. The significant *p* values are in bold.

**Table 2:** Univariate and multivariate analysis of PFS and median OS for untreated NSCLC patients.

**Univariate Analysis**	**PROGRESSION-FREE SURVIVAL**		**OVERALL SURVIVAL**	
	**Hazard Ratio (95% CI)**	***p* Value**	**Hazard Ratio (95% CI)**	***p* Value**
Age (≥65 vs. <65)	1.351 (0.835–2.187)	0.220	1.028 (0.624–1.696)	0.912
Gender (Male vs. Female)	1.190 (0.633–2.237)	0.386	1.320 (0.669–2.607)	0.424
Histology (SQ vs. Non-SQ)	1.345 (0.828–2.183)	0.231	1.286 (0.782–2.114)	0.322
EGFR status (WT vs. Mutant)	1.657 (0.383–7.164)	0.499	2.707 (0.591–12.404)	0.200
Stage (IV vs. III)	**2.223 (1.265–3.908)**	**0.005**	1.176 (0.689–2.007)	0.552
Exosomes (high vs. low)	**3.074 (1.856–5.089)**	**<0.0001**	**3.755 (2.215–6.365)**	**<0.0001**
**Multivariate Analysis**	**PROGRESSION-FREE SURVIVAL**		**OVERALL SURVIVAL**	
	**Hazard Ratio (95% CI)**	***p* Value**	**Hazard Ratio (95% CI)**	***p* Value**
Stage (IV vs. III)	**1.955 (1.113–3.435)**	**0.020**	N/A	N/A
Exosomes (high vs. low)	**2.852 (1.714–4.744)**	**<0.0001**	**3.755 (2.215–6.365)**	**<0.0001**

SQ: squamous; CI: confidence intervals; WT: wild-type; N/A: not applicable. The significant *p* values are in bold.

## Data Availability

The data presented in this study are available on request from the corresponding author. The data are not publicly available.

## Supplementary Materials

The following are available online at https://www.mdpi.com/article/10.3390/cancers13092041/s1, Figure S1: Uncropped Western blot images.
