# Supplementary material for: Small Extracellular Vesicles in Pre-Therapy Plasma Predict Clinical Outcome in Non-Small-Cell Lung Cancer Patients
Source: Cancers (Basel). 2021 Apr 23;13(9):2041. doi: 10.3390/cancers13092041 (PMC8122966; doi:10.3390/cancers13092041)
Supplement: Supplementary file 1 [file cancers-13-02041-s001.zip › cancers-1154655-supplementary.pdf]

Original images of blots and densitometric analysis

Figure 1C

<u>Each lane: 10 μg sEV protein of each patient</u>

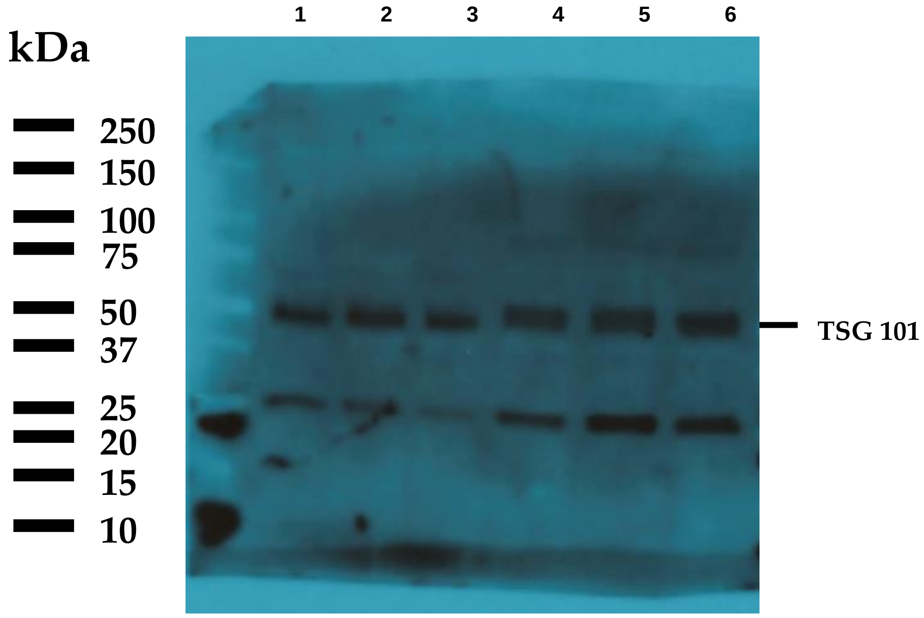

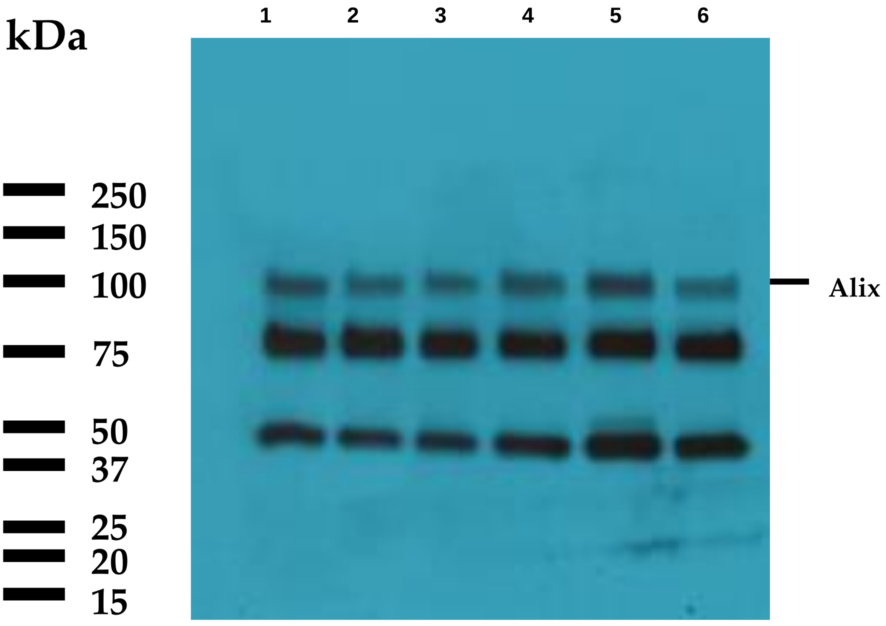

Densitometry analysis

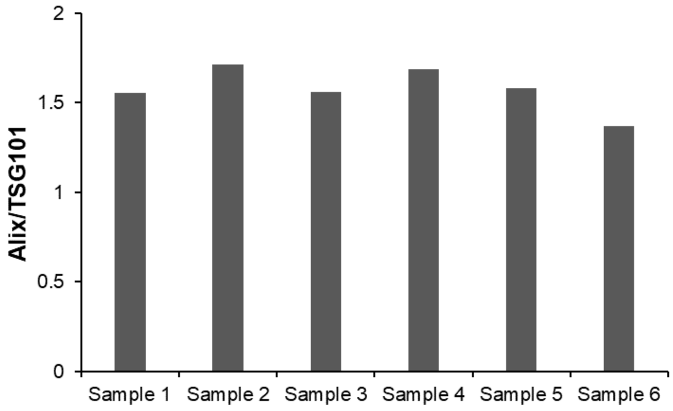

Original images of blots

Figure 2A

<u>Each lane: 10 μg sEV protein of patient (Pt) or healthy donor (HD)</u>

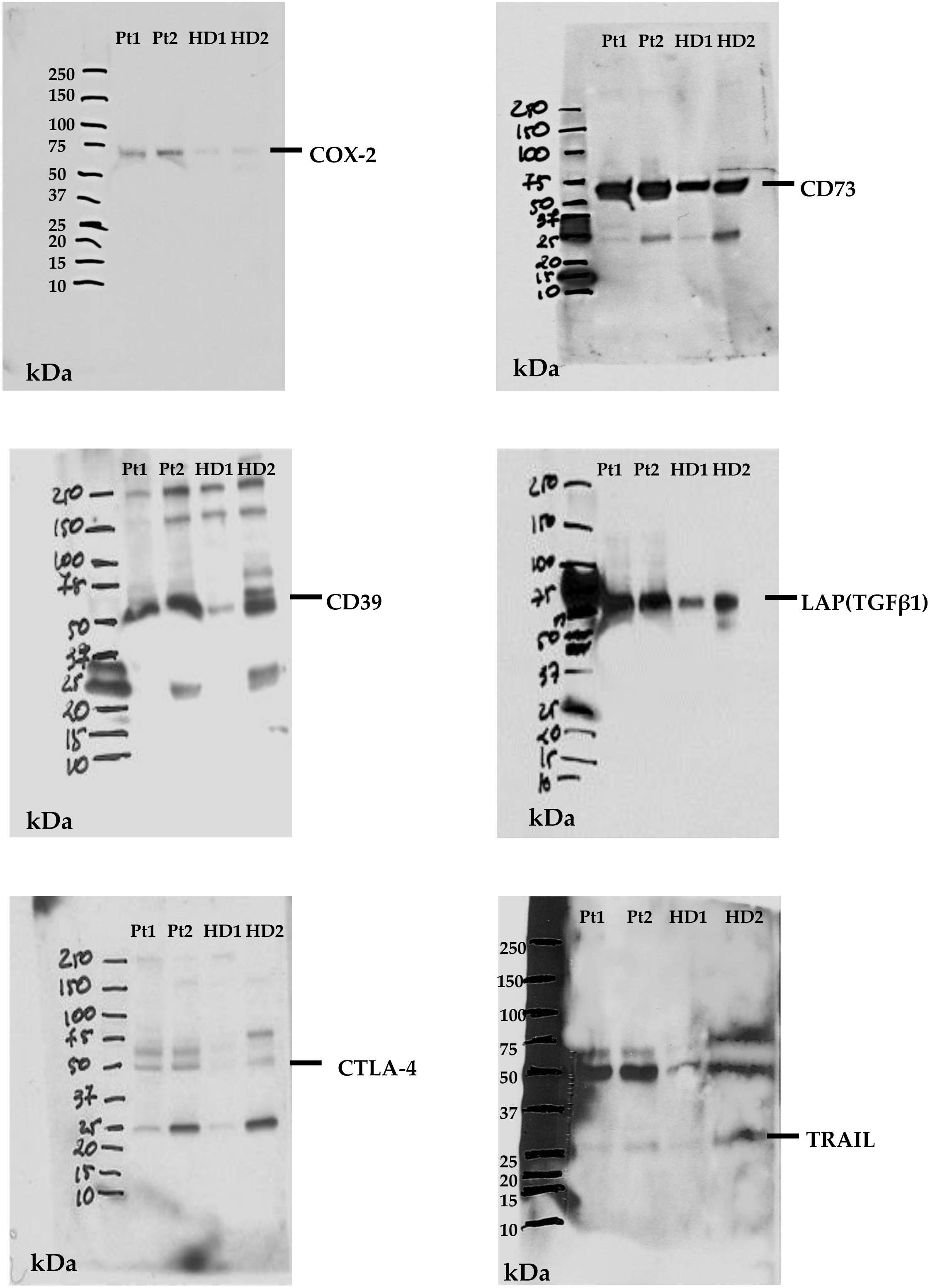

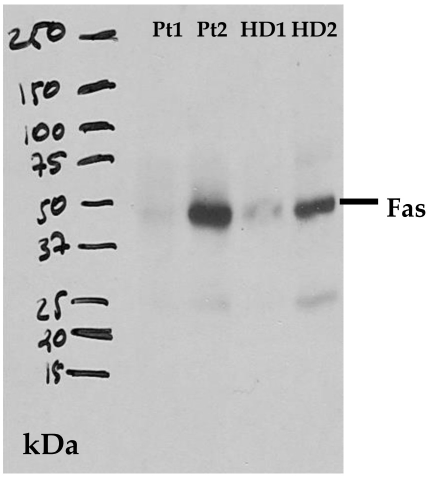
Pt1 Pt2 HD1 HD2
250
150
100
75
50
37
25
20
15
Fas
kDa

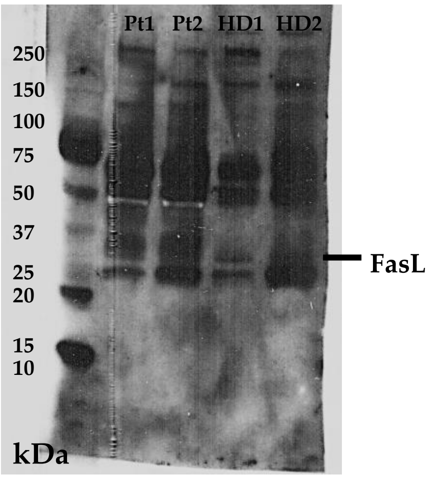
Pt1 Pt2 HD1 HD2
250
150
100
75
50
37
25
20
15
10
FasL
kDa

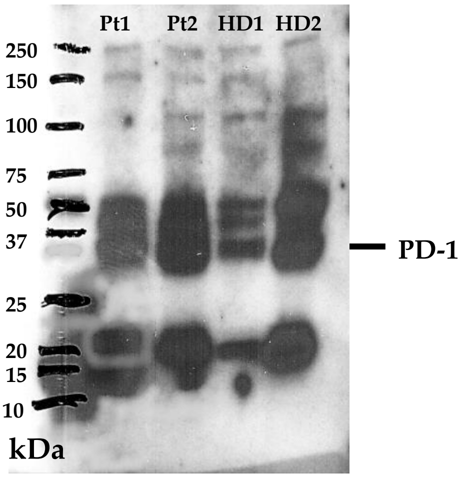
Pt1 Pt2 HD1 HD2
250
150
100
75
50
37
25
20
15
10
PD-1
kDa

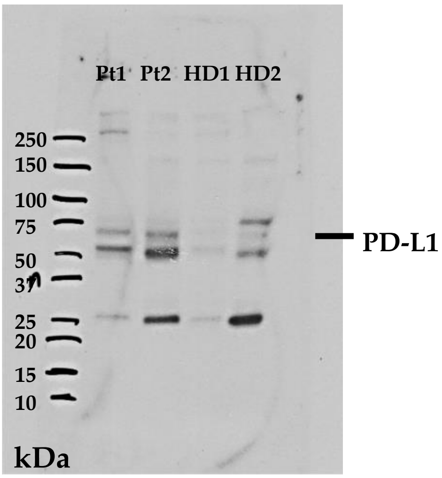
Pt1 Pt2 HD1 HD2
250
150
100
75
50
37
25
20
15
10
PD-L1
kDa

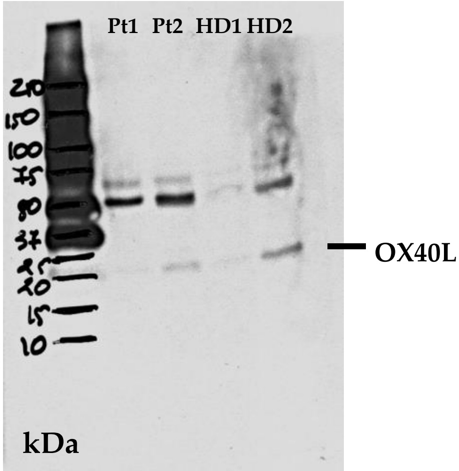
Pt1 Pt2 HD1 HD2
250
150
100
75
50
37
25
20
15
10
OX40L
kDa

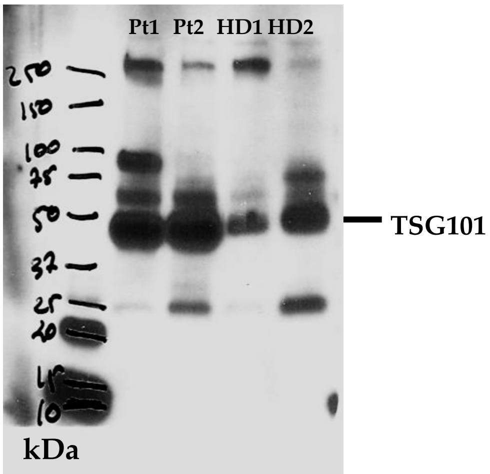
Pt1 Pt2 HD1 HD2
250
150
100
75
50
37
25
20
15
10
TSG101
kDa
